# The effects of environmental enrichment on voluntary physical activity and muscle mass gain in growing rats

**DOI:** 10.3389/fphys.2023.1265871

**Published:** 2023-09-28

**Authors:** Mizuki Sudo, Yutaka Kano, Soichi Ando

**Affiliations:** ^1^ Physical Fitness Research Institute, Meiji Yasuda Life Foundation of Health and Welfare, Tokyo, Japan; ^2^ Graduate School of Informatics and Engineering, The University of Electro-Communications, Chofu, Japan

**Keywords:** physical activity level, environmental enrichment, skeletal muscle, hypertrophy, slow-twitch muscle fiber

## Abstract

**Introduction:** Environmental enrichment (EE) for rodents involves housing conditions that facilitate enhanced sensory, cognitive, and motor stimulation relative to standard housing conditions. A recent study suggested that EE induces muscle hypertrophy. However, it remains unclear whether muscle hypertrophy in EE is associated with voluntary physical activity, and the characteristics of muscle adaptation to EE remain unclarified. Therefore, this study investigated whether muscle adaptation to EE is associated with voluntary physical activity, and assessed the changes in the muscle fiber-type distribution and fiber-type-specific cross-sectional area in response to EE.

**Methods:** Wistar rats (6 weeks of age) were randomly assigned to either the standard environment group (n = 10) or the EE group (n = 10). The voluntary physical activity of rats housed in EE conditions was measured using a recently developed three-axis accelerometer. After exposure to the standard or enriched environment for 30 days, the tibialis anterior, extensor digitorum longus, soleus, plantaris, and gastrocnemius muscles were removed and weighed. Immunohistochemistry analysis was performed on the surface (anterior) and deep (posterior) areas of the tibialis anterior and soleus muscles.

**Results and discussion:** The EE group showed increased voluntary physical activity during the dark period compared with the standard environment group (*p* = 0.005). EE induced muscle mass gain in the soleus muscle (*p* = 0.002) and increased the slow-twitch muscle fiber cross-sectional area of the soleus muscle (*p* = 0.025). EE also increased the distribution of high-oxidative type IIa fibers of the surface area (*p* = 0.001) and type I fibers of the deep area (*p* = 0.037) of the tibialis anterior muscle. These findings suggest that EE is an effective approach to induce slow-twitch muscle fiber hypertrophy through increased daily voluntary physical activity.

## 1 Introduction

Environmental enrichment (EE) for rodents involves housing conditions that facilitate enhanced sensory, cognitive, and motor stimulation relative to standard housing conditions (Nithianantharajah and Hannan, 2006). A large number of studies have suggested that EE conditions have neuroprotective effects on a range of brain functions (van Praag et al., 2000; Kempermann et al., 2002; Mohammed et al., 2002; Nithianantharajah and Hannan, 2006; Amaral et al., 2008; Nag et al., 2009; Viola et al., 2010; Kobilo et al., 2011; Hosseiny et al., 2015; Leger et al., 2015; Li et al., 2015; Mahati et al., 2016; Tomiga et al., 2016). Furthermore, a recent study has shown that EE comprising wheel running and ladders to promote voluntary physical activity also induces muscle hypertrophy (Rostami et al., 2022). This suggests that voluntary physical activity is important in inducing muscle hypertrophy in EE. However, previous studies have reported that EE either did not change the level of voluntary physical activity (Nag et al., 2009) or reduced the voluntary physical activity (Leger et al., 2015). Therefore, it remains unclear whether muscle hypertrophy is linked to voluntary physical activity in EE.

Until recently, voluntary physical activity has not been directly examined using an EE paradigm, primarily due to technical difficulties involved in housing rats in groups. In the present study, we recorded the voluntary physical activity of each rat housed in EE conditions using the recently developed Nano-Tag device, and examined the effects of voluntary physical activity on muscle adaptation to EE. The Nano-Tag device is implanted subcutaneously and records data individually (Yoshizawa et al., 2019; Funabashi et al., 2022). The advantage of the Nano-Tag device is that specialized cages or devices are not required to measure voluntary physical activity in free-moving conditions, which allows the measurement of the individual voluntary physical activity of multiple animals in the same cage without any constraints. An evaluation of the voluntary physical activity of each rat housed in groups in EE conditions will aid in the understanding of the link between voluntary physical activity and muscle hypertrophy in EE. EE is thought to facilitate motor stimulation intermittently, but for a long period. Thus, we hypothesized that the effects of voluntary physical activity are specific to slow-twitch muscle fibers.

The purpose of this study was to investigate whether muscle adaptation to EE is associated with voluntary physical activity. We also assessed the muscle fiber-type distribution and fiber-type-specific cross-sectional area (CSA) to characterize the effects of motor stimulation in EE conditions. This study extends our knowledge of the beneficial effects of EE on voluntary physical activity and provides insight into the association between daily voluntary physical activity and muscle adaptation.

## 2 Materials and methods

### 2.1 Animals and experimental design

All animal care conditions and protocols were approved by the Physical Fitness Research Institute Meiji Yasuda Life Foundation of Health and Welfare Animal Care and Use Committee (permit no. 2014002). Male Wistar rats (6 weeks of age; Japan SLC, Shizuoka, Japan) were housed in a temperature-controlled room (22°C ± 2°C) with a 12-h/12-h light/dark cycle and received standard rat chow and water *ad libitum*. Body weight and food intake were measured weekly.

Rats were randomly assigned to either the standard environment (SE) group or the EE group, and were housed for 30 days. In the SE group, 10 rats were housed in groups in standard laboratory cages (length × width × height: 40 × 25 × 20 cm). In the EE group, 10 rats were housed in groups in large cages (length × width × height: 60 × 40 × 40 cm, which is a 4.8-fold increase compared with the SE group cage volume) to provide extra space for the objects (slope, small hut, tunnels, and running wheel) associated with EE. The objects were changed once every week to maintain novelty.

### 2.2 Measurement of voluntary physical activity

The voluntary physical activity of each rat was recorded using three-axis accelerometers (Nano-Tag: 15 × 14.2 × 7.1 mm, 2.5 g, Kissei Comtec. Co. Ltd., Nagano, Japan). Voluntary physical activity data were obtained from seven rats in each group. Count data were the number of times that the XYZ acceleration vector-synthesized waveform exceeded the threshold. In this study, the threshold was determined during feeding behavior. This allowed the detection of all kinds of movements in the cage as voluntary physical activity. The Nano-Tag was subcutaneously implanted in the back under anesthesia with isoflurane inhalation (2%) and records data individually (Yoshizawa et al., 2019; Funabashi et al., 2022). The Nano-Tag allows the measurement of the voluntary physical activity of multiple free-moving rats without any constraints. Count data were collected using a designated card reader, and voluntary physical activity was assessed every 5 min during the dark and light periods, respectively.

### 2.3 Immunohistochemical analysis

After exposure to either the standard or enriched environment for 30 days, the rats were anesthetized and the tibialis anterior (TA), extensor digitorum longus, soleus (Sol), plantaris, and gastrocnemius muscles were carefully removed and immediately weighed. The TA contains both white (anterior) and red (posterior) muscle, which allows to compare the effects of voluntary physical activity on fast-versus slow-twitch muscle fibers within the same muscle. Thus, we performed immunohistochemical analyses of the surface (anterior) and deep (posterior) areas of the TA. The Sol muscle contains predominantly slow-twitch fibers (Kawano et al., 2008; Larson et al., 2019). Based on the hypothesis that the effects of voluntary physical activity are specific to slow-twitch muscle fibers, we also performed immunohistochemical analyses of the Sol muscle. We expected that these analyses would clarify the characteristics of muscle hypertrophy induced by voluntary physical activity.

Muscle tissue blocks of the TA and Sol muscles were rapidly frozen in isopentane cooled by liquid nitrogen. Transverse sections of 10 µm were made with a cryostat (CM 1950; Leica Biosystems, Jena, Germany) at −20°C and mounted on polylysine-coated slides (Sudo and Kano, 2009). For the TA muscle, to avoid sampling bias, each section was subsampled at four different regions: 1) anterior-medial, 2) anterior-central, 3) posterior-medial, and 4) posterior-central; all regions were analyzed in the respective muscle. For the immunohistochemical assessment of the muscle fiber-type distribution and fiber-type-specific CSA, slides were incubated for 1 h at room temperature in primary antibodies against myosin heavy chain type I (mouse IgG, 1:40, Developmental Studies Hybridoma Bank (DSHB), University of Iowa), type IIa (mouse IgG, 1:1000, DSHB, University of Iowa), type IIb (mouse IgG, 1:100, DSHB, University of Iowa), and type IIx (mouse IgG, 1:100; BF-F3, DSHB, University of Iowa). The sections were allowed to warm to room temperature and were then incubated in phosphate buffer saline (pH 7.5) at 25°C before further incubation with the primary antibody in a humidified box overnight at 4°C. The immunohistochemical reaction was revealed using the Vectastain ABC kit (Vector Laboratories, Funakoshi, Japan) in accordance with the manufacturer’s instructions. Images were visualized with a microscope at ×10 magnification (Nikon) and captured with a digital CCD camera (Cat. No. DP70; Olympus, Tokyo, Japan). The number and CSA of the muscle fibers were measured by tracing the outlines of 50–440 fibers. An NIH imaging software program (ImageJ) was used to determine the CSA.

### 2.4 Statistical analysis

All statistical analyses were performed using Prism version 6.01 (GraphPad Software, San Diego, CA). Group differences in voluntary physical activity and food intake data were determined by a two-way analysis of variance and Tukey-Kramer *post hoc* test (for comparisons between all means); the two-tailed unpaired Student’s t-test (for comparisons of two groups with equal variances) or Welch’s *t*-test (for comparisons of two groups with different variances) were used to compare body weight, muscle weight, CSA, and histological data. In all analyses, *p* < 0.05 was taken to indicate statistical significance. Data are reported as the mean ± standard deviation.

## 3 Results

### 3.1 Body and muscle weights, and food intake

The mean body weight did not differ between the SE and EE groups at the end of the intervention (SE: 288.7 ± 13.2 g, EE: 277.4 ± 13.0 g, *p* = 0.096). Table 1 summarizes the muscle weights. The only muscle weight that differed between the two groups was the Sol weight, which was greater in the EE group than the SE group (*p* = 0.002) (Table 1). There were no differences in weekly food intake between the SE and EE groups (*p* = 0.654) (Table 2).

**TABLE 1:** Muscle weight normalized to body weight in the standard environment (SE) and environmental enrichment (EE) groups.

Muscle	SE group	EE group	*p*-value
Tibialis anterior	1.601 ± 0.075	1.628 ± 0.073	0.456
Extensor digitorum longus	0.411 ± 0.019	0.432 ± 0.032	0.112
Soleus	0.364 ± 0.026	0.428 ± 0.034***	0.002
Plantaris	0.909 ± 0.041	0.936 ± 0.031	0.221
Gastrocnemius	4.789 ± 0.210	4.939 ± 0.167	0.113

Values are given as muscle weight/body weight (mg/g). N = 6–7 in each group. ***p < 0.05.

**TABLE 2:** Food intake in the standard environment (SE) and environmental enrichment (EE) groups.

Food intake	SE group	EE group
Week 1	131 ± 44	144 ± 9
Week 2	136 ± 46	142 ± 18
Week 3	142 ± 49	148 ± 20
Week 4	142 ± 49	162 ± 21

Values are given as food intake (g). N = 10 in each group.

### 3.2 Voluntary physical activity

**FIGURE 1:**
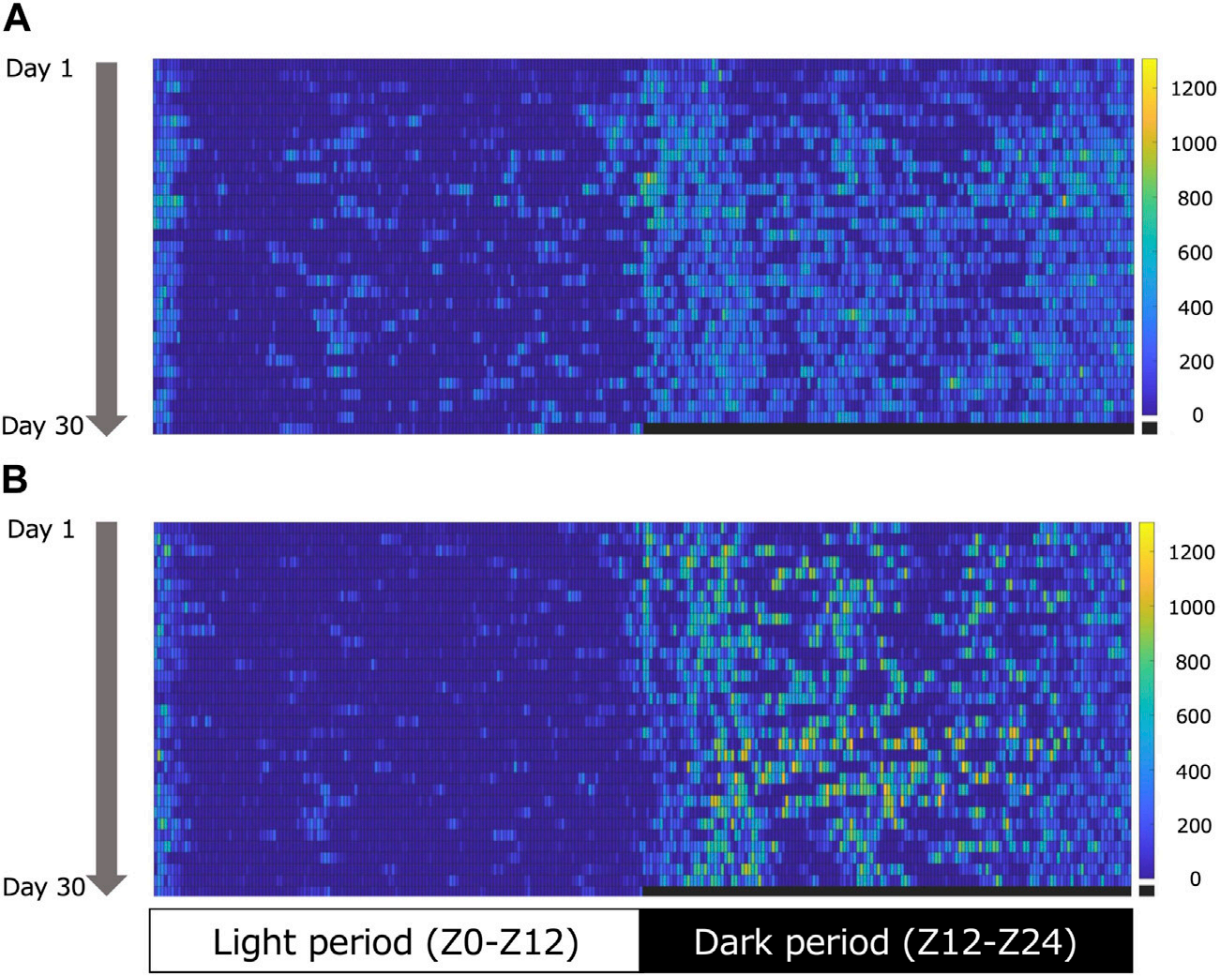
Representative heatmap of voluntary physical activity during the light (left) and dark (right) periods in the standard environment (SE) **(A)** and environmental enrichment (EE) **(B)** groups. The colored pixels in the heat map represent the voluntary physical activity in 5 min. The vertical axis shows the day. The colored bars indicate the range of voluntary physical activity. The lowest voluntary physical activity is displayed in dark blue, and the highest is displayed in yellow.

**FIGURE 2:**
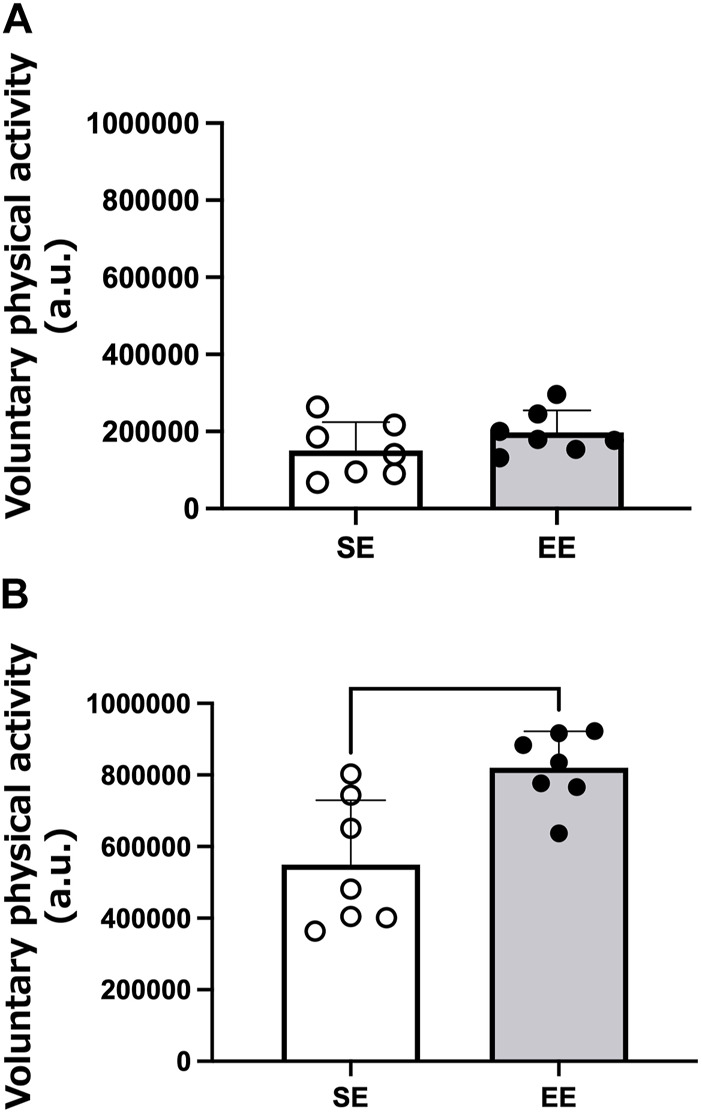
Mean voluntary physical activity of the standard environment (SE) and environmental enrichment (EE) groups during the light **(A)** and dark **(B)** periods. **p < 0.01 *versus* the SE group. N = 7 in each group.

Figure 1 shows an example of the heatmap visualization of voluntary physical activity. Voluntary physical activity was very low during the light period in both groups. Voluntary physical activity increased during the dark period, particularly in the EE group. Figure 2 shows the mean voluntary physical activity in the SE and EE groups. Voluntary physical activity did not differ between the two groups in the light period (*p* = 0.207), but was higher in the EE group than the SE group in the dark period (*p* = 0.005).

### 3.3 Fiber-type distribution and fiber-type-specific CSA

**FIGURE 3:**
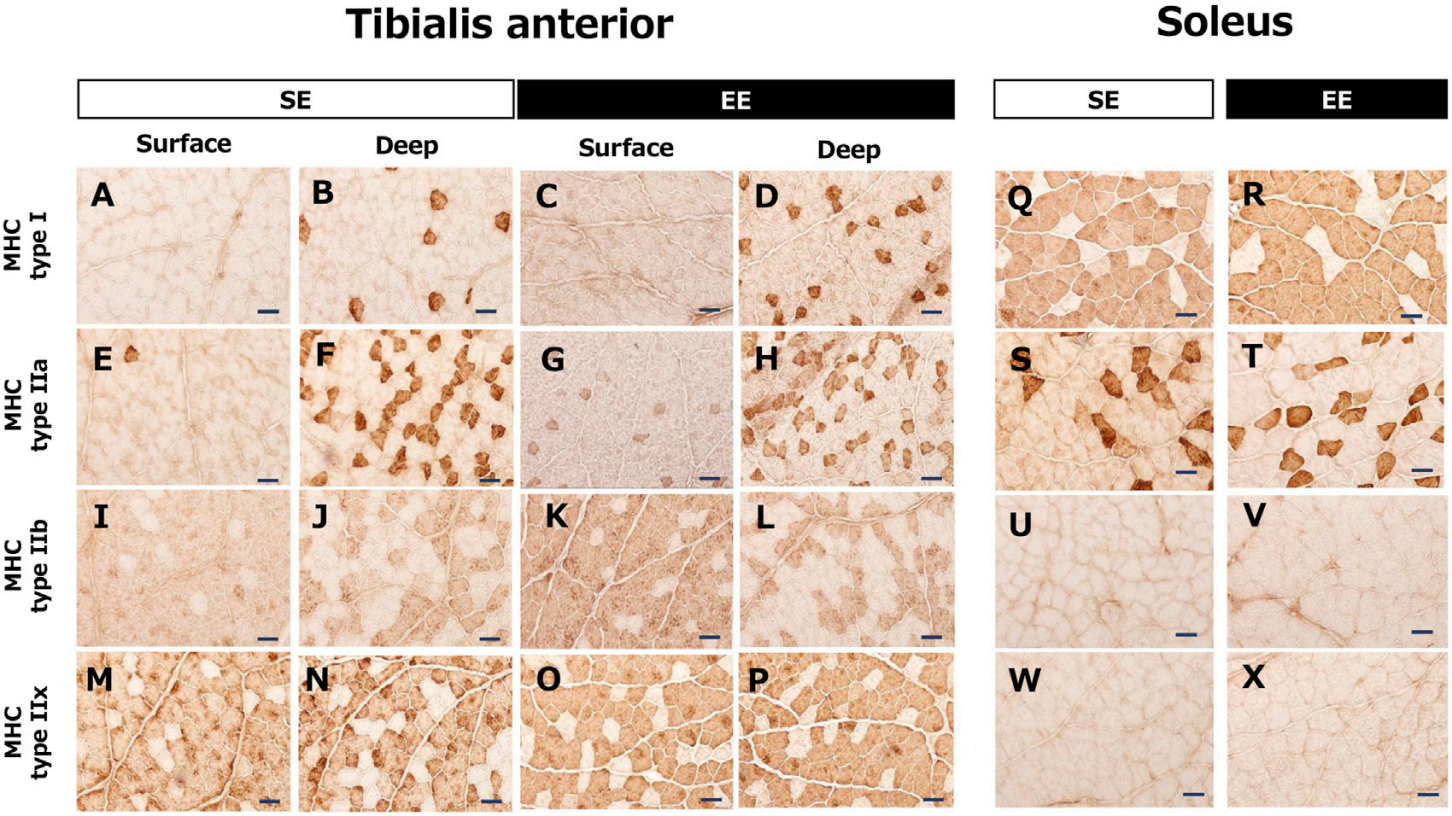
Representative images of the tibialis anterior muscle **(A–P)** and the soleus muscle **(Q–X)** showing myosin heavy chain (MHC) expression in the standard environment (SE) and environmental enrichment (EE) groups. Note that the tibialis anterior muscle includes both white (surface) and red (deep) muscle areas. Bar represents 50 μm.

Figure 3 contains representative images of myosin heavy chain expression in the TA and Sol muscles in the SE and EE groups. Type IIb and type IIx fibers were dominant in the surface area of the TA muscle. In contrast, type I and type IIa fibers were also observed in the deep area of the TA muscle. In the Sol muscle, slow-twitch muscle fibers were dominant, and type IIa fibers were also observed. Staining showed no myosin heavy chain types IIb and IIx in the Sol muscle.

**FIGURE 4:**
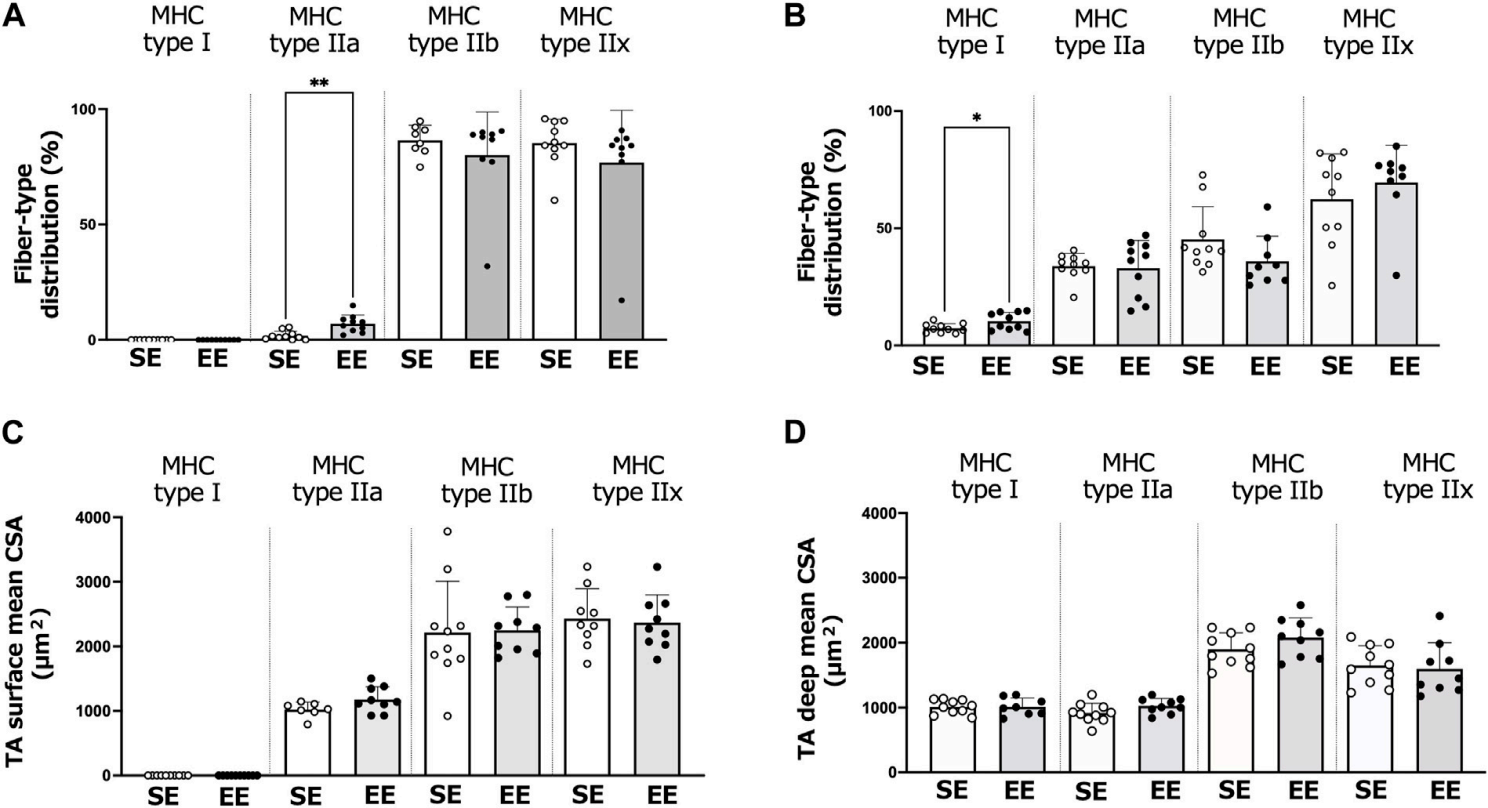
Changes in the tibialis anterior (TA) muscle fiber and size in the standard environment (SE) and environmental enrichment (EE) groups. Muscle fiber-type distribution of the surface **(A)** and deep **(B)** areas of the TA muscle. Surface **(C)** and deep **(D)** areas of the TA muscle fiber cross-sectional area (CSA). Values are shown as means ± standard deviations. **p < 0.01, *p < 0.05 *versus* the SE group. N = 7–10 in each group.

Figure 4 shows the muscle fiber-type distributions and CSA of the surface and deep areas of the TA muscle in the SE and EE groups. In the surface area, the distribution of type IIa fibers was greater in the EE group than the SE group (*p* = 0.001). In the deep area, the distribution of type I fibers was greater in the EE group than the SE group (*p* = 0.037). We observed no differences between the SE and EE groups in the distributions of the other fiber types (*p* > 0.083). There were no differences between the SE and EE groups in the fiber-type specific CSA of the TA muscle.

**FIGURE 5:**
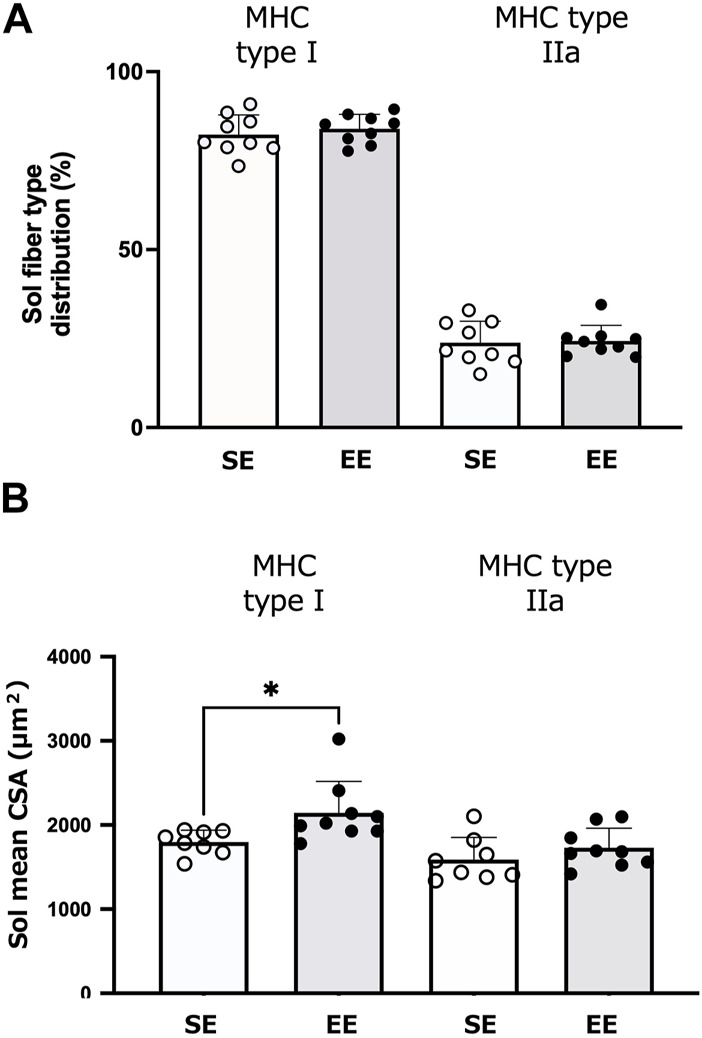
Changes in the soleus (Sol) muscle fiber and size in the standard environment (SE) and environmental enrichment (EE) groups. Muscle fiber-type distribution of the Sol muscle **(A)** and the Sol fiber cross-sectional area (CSA) **(B)**. Values are shown as means ± standard deviations. *p < 0.05 *versus* the SE group. N = 8–9 in each group.

Figure 5 depicts the muscle fiber-type distributions and CSA of the Sol muscle in the SE and EE groups. There were no differences between groups in the distributions of type I (*p* = 0.477) and type IIa fibers (*p* = 0.836). The CSA of slow-twitch fibers was larger in the EE group than the SE group (*p* = 0.025). The CSA of type IIa fibers did not differ between groups (*p* = 0.267).

## 4 Discussion

The EE paradigm has been used to examine behavioral modifications and neuroplasticity by increasing voluntary physical activity, learning experiences, visual inputs, and social interactions (van Praag et al., 2000; Kempermann et al., 2002; Mohammed et al., 2002; Nithianantharajah and Hannan, 2006; Amaral et al., 2008; Nag et al., 2009; Viola et al., 2010; Kobilo et al., 2011; Hosseiny et al., 2015; Leger et al., 2015; Li et al., 2015; Mahati et al., 2016; Tomiga et al., 2016). A recent study suggested that EE also induces muscle hypertrophy via enhanced voluntary physical activity (Rostami et al., 2022); however, the authors did not directly measure the voluntary physical activity. Hence, we directly measured the voluntary physical activity of each rat and investigated whether muscle hypertrophy was accompanied by increased voluntary physical activity. The novel findings of the present study are: 1) muscle hypertrophy was linked with increased voluntary physical activity under EE conditions; and 2) muscle hypertrophy in the EE condition was specific to the Sol muscle and slow-twitch muscle fibers.

In previous studies, voluntary physical activity has been measured indirectly using an infrared light or sensor (Nag et al., 2009; Leger et al., 2015; Yamada et al., 2015; Morimoto et al., 2017). However, these measurements are not appropriate to assess the voluntary physical activity of each animal for long durations when animals are housed in groups. To circumvent this problem, we used the recently developed Nano-Tag device to assess the voluntary physical activity of each rat housed in groups. It is well known that rodents are more active during the dark period compared with the light period (Roedel et al., 2006). As expected, voluntary physical activity was higher during the dark period in the EE group than in the SE group in the present study, indicating that EE enhanced voluntary physical activity during the dark period. Conversely, there were no differences between the SE and EE groups in voluntary physical activity during the light period, indicating that EE did not increase the voluntary physical activity of rats during the period when they are generally inactive. As muscle hypertrophy in the EE condition was accompanied by increased voluntary physical activity during the dark period, this suggests that the observed muscle hypertrophy is primarily ascribed to increases in voluntary physical activity during the dark period in rats provided with EE.

As shown by the heatmap (Figure 1), voluntary physical activity was intermittent but lasted for a long time during the dark period. Furthermore, the present EE condition included the running wheel. Therefore, although we were not able to evaluate the intensity of voluntary physical activity in the EE condition, we can assume that the voluntary physical activity in the EE condition was mainly endurance exercise. A recent study indicated that 3 weeks of endurance exercise on a treadmill induces hypertrophy of the Sol muscle in rats (Yin et al., 2020). Furthermore, EE increases satellite cell numbers, together with paired box transcription factor 7 and myogenic determination protein mRNA expression in the flexor hallucis longus muscle of rats (Rostami et al., 2022). Therefore, the present results suggest that daily voluntary physical activity in the EE condition has the potential to induce muscle hypertrophy.

Muscle hypertrophy was exclusively observed in the Sol muscle in the EE group, and increases in the CSA were specific to slow-twitch muscle fibers. The Sol is an antigravity muscle that has a high proportion of slow-twitch fibers (Kawano et al., 2008; Larson et al., 2019). Slow- and fast-twitch muscle fibers have specific contractile properties to respond to specific needs (Desaphy et al., 2001). In the present EE condition, in addition to running in a wheel, the rats often stood upright or climbed objects. Repeated muscle contractions in the EE condition (e.g., running, standing, and climbing) were likely to induce hypertrophy of the Sol muscle. In contrast to voluntary physical activity, a slow-to-fast transition occurs together with atrophy in the antigravity slow-twitch Sol muscle after periods of rat hindlimb unloading (Desaphy et al., 2001). This implies that the Sol muscle is susceptible to changes in motor stimulation. Hence, it is reasonable that enhanced voluntary physical activity specifically affected the Sol muscle and slow-twitch muscle fibers.

Type IIa fibers are fast-twitch fibers that have high oxidative and glycolytic capacity and are relatively resistant to fatigue (Herbison et al., 1982). In the present study, we observed increases in the distribution of type IIa fibers in the surface area of the TA muscle in the EE group, suggesting that changes in the muscle fiber-type distribution of the TA muscle were limited to high-oxidative muscle fibers that have intermediate characteristics between fast- and slow-twitch fibers. The distribution of type I fibers in the deep area of the TA muscle also increased in the EE group. These results are compatible with the notion that voluntary physical activity predominantly affects slow-twitch muscle fibers. Collectively, these findings suggest that muscle hypertrophy in the EE condition is characterized by specific effects on slow-twitch or high-oxidative muscle fibers.

In contrast to our study, Nag et al. (2009), indicated that EE does not alter voluntary physical activity in juvenile mice, and Leger et al. (2015) reported that the voluntary physical activity of mice is reduced in the EE condition. These findings are inconsistent with the present results showing that EE increased voluntary physical activity. However, there are two possible explanations for the discrepancies. First, the housing conditions differed between the present study and the two previous studies. In general, EE consists of a complex housing condition that increases the opportunities for voluntary physical activity, learning, and socialization. Hence, it is plausible that housing conditions determine whether EE increases voluntary physical activity. In the present study, the EE cages contained a slope, a small hut, three tunnels, and a running wheel to promote voluntary physical activity. Hence, motor stimulation was likely to dominate in the present EE condition, which resulted in increased voluntary physical activity. Additionally, the duration of measurement in the present study was far longer (30 days) than the durations in the two previous studies. The differences in measurement duration may also be associated with the differences in observed voluntary physical activity.

There are a few limitations that warrant consideration. First, given the age of the rats when the intervention was started (6 weeks of age), it is premature to conclude that the present observations are solely ascribed to voluntary physical activity. For example, Larson et al. (2019) has shown that increases in body and muscle weights of normal rats are prominent up to the first 6 weeks, followed by secondary increases afterwards. Tamaki and colleagues also indicated that muscle weight and CSA increase up to 10 weeks of age (Tamaki et al., 2002). Thus, although the Sol muscle weight normalized to body weight was substantially greater in the EE condition as compared with SE condition (Table 1), we cannot detect whether the observed muscle hypertrophy is due to physical activity or growth acceleration. Additional measurements of growth parameters (e.g., tibia length) would be necessary to conclude that the observed muscle hypertrophy is associated with voluntary physical activity. Second, we did not evaluate the CSA of slow-twitch fibers in the same planter flexor muscle (i.e., plantaris and gastrocnemius muscles). Although muscle weights of these muscles did not increase in the EE condition, the analyses may provide additional insights into the muscle adaptation in the EE condition. Finally, we did not assess muscle protein synthesis and mitochondrial and capillary adaptations in the EE condition. Further investigation would be necessary to understand muscle adaptations to environmental enrichment.

## 5 Conclusion

The present study used a recently developed three-axis accelerometer to assess the voluntary physical activity of rats housed in either the SE or EE condition, and examined the association between voluntary physical activity and muscle hypertrophy in the EE condition. Muscle hypertrophy was accompanied by increases in voluntary physical activity. EE induced muscle hypertrophy of the Sol and increased the CSA of slow-twitch muscle fibers of the Sol. EE also increased the distribution of high-oxidative type IIa fibers in the surface area and type I fibers in the deep area of the TA muscle. These findings suggest that EE is an effective approach to induce slow-twitch muscle fiber hypertrophy through increased daily voluntary physical activity. The selective hypertrophy of slow-twitch muscle fibers in the EE condition may involve a specific adaption, including increased endurance and oxidative capacity, to relatively low, but repetitive, motor stimuli associated with voluntary physical activity.

## Data Availability

The original contributions presented in the study are included in the article/Supplementary material, further inquiries can be directed to the corresponding author.
